# Obstetrics—the Science and Art

**Published:** 1857-01

**Authors:** 


### BOOK NOTICES.

*Obstetrics — the Science and Art.* By CHARLES D. MEIGS, M.D. Prof. of Midwifery and Diseases of Women and Children, Jefferson Medical College, at Philadelphia, &c. Third Edition Revised. With One Hundred and Twenty-Nine Illustrations. Philadelphia, Blanchard & Lea, 1856.

The first and second editions of this work have been before the profession long enough to be well known: and the fact, that a third edition has been so soon called for, is sufficient evidence of its having been highly appreciated. The present volume contains seven hundred and fifty-eight pages, well printed, and substantially bound in calf. It has been carefully revised by the author, and is the best American work on Midwifery that is accessible to the student and practitioner.

